# Composite Poly(vinyl alcohol)-Based Nanofibers Embedding Differently-Shaped Gold Nanoparticles: Preparation and Characterization

**DOI:** 10.3390/polym13101604

**Published:** 2021-05-16

**Authors:** Andrea Dodero, Maila Castellano, Paola Lova, Massimo Ottonelli, Elisabetta Brunengo, Silvia Vicini, Marina Alloisio

**Affiliations:** 1 Department of Chemistry and Industrial Chemistry, Università degli Studi di Genova, Via Dodecanso 31, 16146 Genoa, Italy; maila.castellano@unige.it (M.C.); paola.lova@unige.it (P.L.); massimo.ottonelli@unige.it (M.O.); silvia.vicini@unige.it (S.V.); 2 Institute of Chemical Sciences and Technologies “Giulio Natta” (SCITEC), Italian National Research Council (CNR), Via De Marini 6, 16149 Genova, Italy; elisabetta.brunengo@scitec.cnr.it

**Keywords:** gold nanoparticles, surface plasmon resonance, optical properties, electrospinning, nanofibers, poly(vinyl alcohol), composites, thermal properties

## Abstract

Poly(vinyl alcohol) nanofibrous mats containing ad hoc synthesized gold nanostructures were prepared via a single-step electrospinning procedure and investigated as a novel composite platform with several potential applications. Specifically, the effect of differently shaped and sized gold nanostructures on the resulting mat physical-chemical properties was investigated. In detail, nearly spherical nanoparticles and nanorods were first synthesized through a chemical reduction of gold precursors in water by using (hexadecyl)trimethylammonium bromide as the stabilizing agent. These nanostructures were then dispersed in poly(vinyl alcohol) aqueous solutions to prepare nanofibrous mats, which were then stabilized via a humble thermal treatment able to enhance their thermal stability and water resistance. Remarkably, the nanostructure type was proven to influence the mesh morphology, with the small spherical nanoparticles and the large nanorods leading to thinner well defined or bigger defect-rich nanofibers, respectively. Finally, the good mechanical properties shown by the prepared composite mats suggest their ease of handleability thereby opening new perspective applications.

## 1. Introduction

The recent advances in nanotechnology have opened the way to the design and fabrication of new materials for a broad variety of applications including the biomedical [1] and pharmaceutical industries [2], the textile [3] and the agriculture sectors [4], food packaging products [5], sensing materials [6,7,8], and wastewater remediation treatments [9]. In this sense, plasmonic nanoparticles are characterized by an increasing deal of interest owing to their unique physical and chemical properties [10]. Specifically, when these materials are excited by electromagnetic radiation of specific wavelengths, the conduction electrons on their surface undergo a collective oscillation (i.e., localized surface plasmon resonance—LSPR) which results in an unusually strong scattering and absorption of light. It is noteworthy that plasmonic nanoparticles display a color that depends on their size and shape, as well as on their composition. Hence, by changing the nanoparticle geometry the overall optical response can be tuned through the visible and near-infrared regions of the electromagnetic spectrum. Despite the fact that plasmonic nanoparticles can be comprised of any metals and even some semiconductors, gold nanoparticles (AuNPs) have drawn the greatest interest due to their ease of fabrication and functionalization, inertness, and very intense color [11,12]. Nevertheless, besides these indisputable advantages that make AuNPs extremely promising for a broad range of purposes (e.g., biological and chemical sensing, diagnostics and medical therapeutics, surface-enhanced Raman scattering, visible light-responsive photocatalysis, surface patterning), their proficient usage is to some extent hindered by several factors. By way of example, AuNPs are usually prepared as suspensions in a specific solvent (e.g., water), which poses not negligible limitations in their easy handleability.

In this regard, the electrospinning technique represents a straightforward manufacturing approach for the fabrication of functionalized nanostructured materials embedding nanoparticles [13,14,15]. Indeed, electrospun nanofibers are nowadays vastly explored in different fields including tissue engineering [16,17,18], drug delivery [19,20], wastewater remediation [21,22], sensing [23], food packaging [24], and textiles [25]. Electrospinning consists of the application of a strong electric field to a polymer solution, or polymer melt, which is usually extruded via a spinneret at a constant rate. When the accumulated surface charge overcomes the liquid surface tension, a continuous polymer jet is ejected from the tip of the spinneret and it moves quickly towards a metallic collector. During this flight, the jet solidifies before being collected on the metallic substrate in the form of a non-woven nanofibrous mat. This unique structure endows electrospun membranes with a very high surface area and enriches them with peculiar physical-chemical properties. Additionally, almost any existing polymer, either synthetic or natural, can be electrospun and the resultant nanofibers can be easily functionalized with tailored properties [26,27,28].

With these premises, it is not surprising that electrospun membranes represent the ideal matrix to embed gold nanoparticles in order to fully exploit their outstanding features. By way of example, such materials have shown great promises as controlled drug delivery systems in biomedical and pharmaceutical products [29], in smart attenuators for technological purposes [30], and even in self-disinfecting personal protective equipment [31]. In the present work, the synthesis of differently shaped and sized AuNPs and their subsequent embedding in electrospun membranes was demonstrated. Firstly, gold nanoparticles were chemically synthesized in water by properly adjusting well-established, bottom-up methods and then characterized by means of morphological and spectroscopic techniques. Then, AuNPs were directly dispersed in poly(vinyl alcohol)-based aqueous formulations, and composite membranes were fabricated via a single-step electrospinning procedure. Before characterization, the prepared mats were subjected to an annealing/stabilization treatment to reduce the polymer solubility in water. Finally, the optical, thermal, morphological, and mechanical properties of the electrospun nanofibers were assessed taking into account both the effects of the annealing process and the nanoparticle presence. In this sense, the choice of AuNPs of various geometries was specifically explored to evaluate the effect of their shape and dimensions on the mat’s overall physical-chemical properties.

## 2. Materials and Methods

### 2.1. Materials

Poly(vinyl alcohol) (PVA) fully hydrolyzed with M_w_ = 89,000–98,000 g/mol was purchased from Sigma-Aldrich (Merck Life Science S.r.l., Milan, Italy). Hydrogen tetrachloroauric acid (HAuCl_4_, Alfa Aesar, Thermo Fisher GmBH, Kandel, Germany), (hexadecyl)trimethylammonium bromide (CTAB, Alfa Aesar, Thermo Fisher GmBH, Kandel, Germany), sodium borohydride (NaBH_4_, Sigma-Aldrich, Merck Life Science S.r.l, Milan, Italy), silver nitrate (AgNO_3_, Alfa Aesar, Thermo Fisher GmBH, Kandel, Germany), and L-ascorbic acid (Sigma-Aldrich, Merck Life Science S.r.l, Milan, Italy) were commercial products used as received.

Aqueous solutions were prepared with ultra-high-purity Milli-Q water distilled twice prior to use. Glassware was thoroughly cleaned before use with fresh “piranha” solution obtained by mixing concentrated sulfuric acid and cooled hydrogen peroxide (30 *v*/*v*%) in the ratio of 2:1 *v/v* and then rinsed with bi-distilled water.

### 2.2. Methods

#### 2.2.1. Synthesis of Gold Nanoparticles

CTAB-stabilized gold nanoparticles of nearly spherical shape (AuNPs 1 and AuNPs 2) were obtained through the wet chemical reduction of HAuCl_4_ by adapting standard protocols. In a thoroughly cleaned flask, 20 mL of bi-distilled water was added to the proper aliquots of aqueous solutions of HAuCl_4_ (100 mmol/L) and CTAB (200 mmol/L) and kept under stirring at room temperature for half an hour. Then, a proper aliquot of 20 min aged, iced solution of NaBH_4_ in water (25 mmol/L) was introduced drop by drop. The procedure was repeated at different reagent concentrations, maintaining the molar ratio between CTAB/HAuCl_4_ and NaBH_4_/HAuCl_4_ equal to 3 and 5, respectively. In detail, 10 µL of HAuCl_4_ (100 mmol/L), 15 µL of CTAB (200 mmol/L), and 200 µL of NaBH_4_ (25 mmol/L) were used for the synthesis of the sample of AuNPs 1, whereas 60 µL of HAuCl_4_ (100 mmol/L), 90 µL of CTAB (200 mmol/L), and 1.2 mL of NaBH_4_ (25 mmol/L) were used for the synthesis of the sample of AuNPs 2. The mixtures, which immediately turned from bright yellow to red, were maintained under constant stirring overnight to ensure full reaction. The crude products, corresponding to aqueous suspensions of nominal concentrations of 0.05 and 0.3 mmol/L in terms of Au content, were then stored at room temperature.

CTAB-coated gold nanorods (AuNPs 3 and AuNPs 4) were instead obtained through a standard, seed-mediated growth method [32] by varying the type of the preformed gold nanoparticles used as seeds for the fabrication of the nanorods. In a typical synthesis, a growth solution was prepared by mixing in a thoroughly cleaned flask bi-distilled water (25 mL), CTAB (25 mL, 200 mmol/L), HAuCl_4_ (0.27 mL, 100 mmol/L), fresh AgNO_3_ (0.14 mL, 30 mmol/L), and fresh L-ascorbic acid (1.6 mL, 10 mmol/L). The mixture was stirred until it became colorless and finally 0.28 mL of the gold seed solution was added under vigorous stirring for 20 s. Specifically, an aliquot of 2.5 h aged AuNPs 2 sample was used as a seed for the preparation of the AuNPs 3 sample, whereas an aliquot of 30-min aged AuNPs 3 sample was used as a seed for the preparation of the AuNPs 4 sample. The resultant mixtures of a nominal concentration of 0.5 mmol/L in terms of Au content turned to a deep blue and brown color, respectively, after few minutes of aging and were stored at room temperature without being purified from CTAB excess.

#### 2.2.2. Preparation and Rheological Characterization of PVA-AuNP Suspensions

To prepare PVA-AuNPs suspensions, a proper amount of the polymer powder was first dissolved in deionized water under magnetic stirring at T = 80 °C for 3 h. Then, the resultant PVA solutions were cool down at room temperature and finally diluted with the appropriate AuNPs suspension. In the final mixtures, the PVA concentration was kept constant at 12.5 *wv*%, whereas the nanoparticle concentration was specifically selected every time in order to achieve the same optical density for each formulation.

PVA-AuNPs suspensions were rheologically characterized by means of an MCR 301 rotational rheometer (Anton Paar GmbH, Graz, Austria) equipped with a Peltier heating system and a solvent trap chamber. The temperature was fixed at T = 25 ± 0.2 °C. Steady-state viscosity measurements were carried out in the shear rate range of 0.01–1000 s^−1^ using a 25 mm plate-plate (PP25) geometry with a 1 mm gap.

#### 2.2.3. Electrospinning and Membrane Thermal Stabilization

To obtain nanofibrous membranes, PVA-AuNPs suspensions were processed via an electrospinning professional machine (Doxa Microfluidics, Malaga, Spain). In a typical experiment, 10 mL of each mixture was electrospun on an aluminum rotating drum collector covered with baking paper using a spinneret-collector distance of 15 cm, an applied voltage of 35 kV, an infuse rate of 0.75 mL/h, a 20 G flat-tip needle, and a collector rotating speed of 100 rpm. Temperature and relative humidity were maintained constant at 25 °C and 50%, respectively. After their preparation, the electrospun membranes were carefully peeled off from the collector and thermally treated at T = 180 °C for 2 h.

#### 2.2.4. Spectroscopic Characterization

UV-vis-NIR spectra of AuNPs suspensions were recorded at room temperature by means of a Shimadzu UV-1800 (Shimadzu USA Manufacturing, Inc., Canby, OR, USA) spectrophotometer with fused silica cuvettes of different path lengths.

Diffuse reflectance measurements were collected on PVA-AuNPs electrospun membranes with a fiber-based set-up coupling an AvaSphere-50 integration sphere (Avantes, Apeldoorn, The Netherlands) to an AvaSpec-ULS2048CL-EVO UV-VIS-NIR detector with a spectral resolution of 1.4 nm (Avantes, Apeldoorn, The Netherlands) and an AvaLight-Mini Halogen light source (Avantes, Apeldoorn, The Netherlands).

#### 2.2.5. Morphological Investigation

AuNPs were explored for their morphology via field-emission scanning electron microscopy (FE-SEM) by using a ZEISS SUPRA 40 VP (Carl Zeiss NST, GmbH, Oberkochen, DEU) microscope operating at 20 keV in backscattered configuration (QBSD mode). Before the analysis, the nanoparticle-containing samples were thinly sputter-coated with carbon using a Polaron E5100 sputter coater (2 M Strumenti, Rome, Italy) to obtain good conductivity.

PVA-AuNPs electrospun membranes were characterized by means of scanning electron microscopy (SEM) via a Hitachi TM3000 benchtop microscope (Hitachi High-Tech GLOBAL, Tokyo, Japan) operating at an acceleration voltage of 5 kV. The sample surface was sputtered with silver using a Quorum Q150R ES sputter coater (2 M Strumenti, Rome, Italy) at 50 mA for 2 min.

ImageJ open-source software (National Institute of Health, Bethesda, MD, USA) was used to measure the size and shape of the gold nanostructures from the FE-SEM images. Afterward, a statistical analysis of the collected data was carried out based on the Kolmogorov–Smirnov [33] and the median absolute deviation (MAD) methods [34].

#### 2.2.6. Thermal Analysis

Differential scanning calorimetry (DSC) was carried out on the electrospun membranes via a DSC1 STARe instrument (Mettler Toledo, Columbus, OH, USA) in the 0–250 °C temperature range using a heating rate of 20 °C/min and a continuous nitrogen flow of 10 mL/min.

Thermogravimetric analysis was performed on the electrospun membranes via a TGA/DSC1 STARe instrument (Mettler Toledo, Columbus, OH, USA). The samples were heated under a continuous nitrogen flow (i.e., 80 mL/min) in the 30–700 °C temperature range and then under a continuous oxygen flow (i.e., 80 mL/min) in the 700–900 °C temperature range. A heating rate of 10 °C/min was always used.

#### 2.2.7. Dynamic-Mechanical Investigation

Dynamic-mechanical analysis (DMA) was carried out on the nanofibrous membranes via an MCR 301 rotational rheometer (Anton Paar GmbH, Graz, Austria) supplied with a universal extensional fixture geometry (UXF) and a CDT-450 chamber. Rectangular specimens (40 mm × 10 mm) were prepared from the electrospun mats by using a punch cutter and each sample thickness was measured via a digital micrometer. The linear viscoelastic region (LVER) of the samples was first explored via amplitude sweep tests (AS) at T = 25 ± 1 °C using a frequency (n) and extensional stress (σ) of 1 Hz and in the range of 0.01–10%, respectively. Then, frequency sweep tests (FS) were carried out at T = 25 ± 1 °C with a fixed σ = 0.1 MPa varying the frequency between 0.01 and 10 Hz. A static stress of 2 MPa was applied for all experiments to ensure correct sample loading and result reliability.

## 3. Results and Discussion

### 3.1. Characterization of CTAB-Stabilized AuNPs

**Figure 1:**
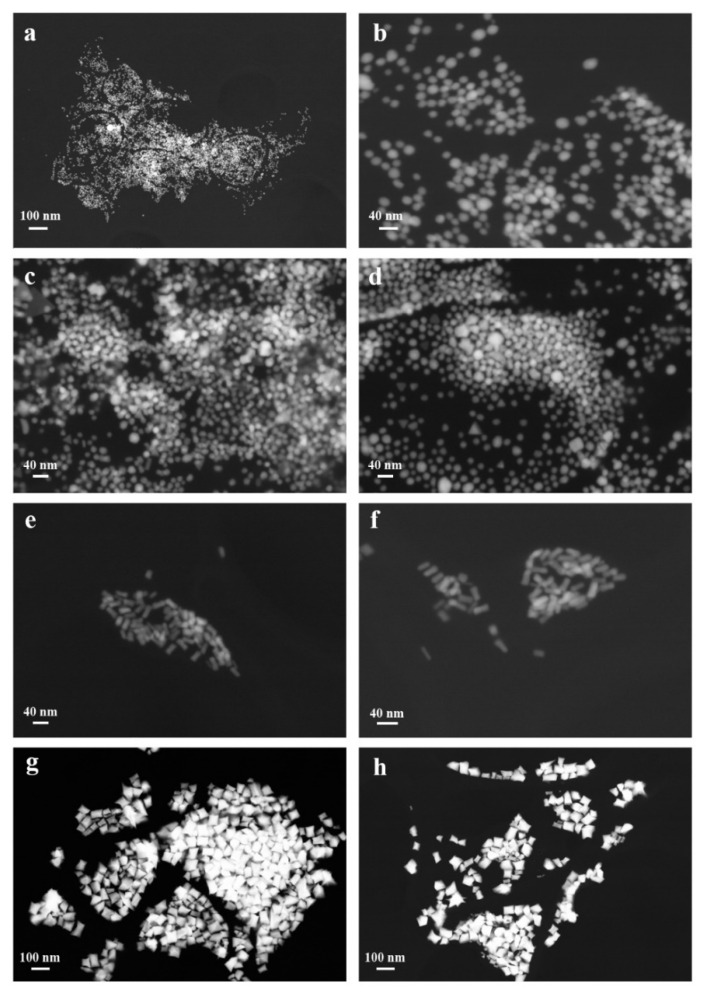
SEM micrographs at two different magnifications for (**a**,**b**) AuNPs 1, (**c**,**d**) AuNPs 2, (**e**,**f**) AuNPs 3, and (**g**,**h**) AuNPs 4 samples.

High-resolution FE-SEM images of gold nanoparticles stabilized with CTAB molecules were acquired in a backscattered configuration to assess their geometry (Figure 1). The dimensions of the samples were measured on more than 200 nanoparticles and reported in Table 1.

As clearly shown in the micrographs of Figure 1a–d, AuNPs 1 and AuNPs 2 are mostly composed of quite homogeneous, spherical particles of average dimensions equal to 13.4 and 15.5 nm, respectively. Although minimal, the size difference between the two samples is related to the different synthetic protocols. On the contrary, the micrographs in Figure 1e–h reveal the presence of anisotropic nanostructures in a very high shape yield (i.e. >90%). In particular, AuNPs 3 is constituted of nanorods with a diameter of about 16 nm, length of about 36 nm, and limited size dispersion. Instead, AuNPs 4 is formed by thicker, more dispersed nanorods of about 70 nm diameter and nearly triple in length with respect to the AuNPs 3 sample (i.e., about 100 nm). Once again, the variations in size and aspect ratio are assigned to the different synthetic conditions, and particularly to the type of seeds used in the sample preparation [35].

**Figure 2:**
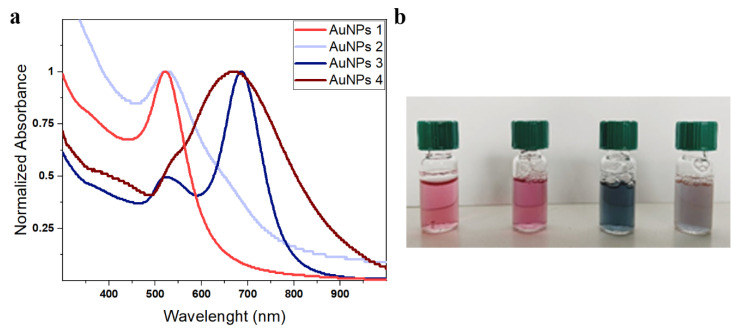
(**a**) Normalized UV-vis spectra of the different AuNPs aqueous suspensions and (**b**) photographs of aqueous suspensions of AuNPs 1, AuNPs 2, AuNPs 3, and AuNPs 4 (from left to right).

The synthesized gold nanoparticles were also spectroscopically investigated in the form of aqueous suspensions. Figure 2 shows the UV-vis-NIR spectra (normalized at the absorbance maximum to facilitate the comparison), whereas the corresponding peak wavelengths are listed in Table 1. Digital photographs of the colloidal suspensions are also reported alongside.

Both AuNPs 1 and AuNPs 2 exhibit an LSPR band peak at around 520 nm, typical of isotropic gold nanostructures. The sharper and slightly blue-shifted line shape of AuNPs 1 compared to the spectrum of AuNPs 2 is in agreement with the smaller size and dispersity of the sample. In the spectral profile of AuNPs 3, two distinct plasmonic resonances, centered at 524 and 686 nm, can be detected. Such features are typical of anisotropic nanostructures. The higher energy band positioned at ~520 nm corresponds to the transversal component of the plasmon resonance with respect to the log-axis of the rods, whereas the lower energy band at around 690 nm is assigned to the longitudinal component. AuNPs 4 shows a broad signal extending from the visible to NIR region and is characterized by the presence of a main peak at around 670 nm and a shoulder at around 550 nm. These features can be attributed to the partial overlap of the LSPR transversal and longitudinal components of gold nanorods of increased size and reduced aspect ratios such as those depicted in Figure 1g,h.

On the whole, the results of the spectroscopic characterization of the AuNP samples are fully consistent with their morphological data.

Further confirmation on the coherence between spectroscopic and morphological data arises from a simulation of the nanoparticle spectral features through a program based on the Mie and Gans (MG) model [36]. The calculated extinction profiles of all samples obtained by employing the size values of Table 1 as fitting parameters provide maximum wavelengths for the LSPR transversal components between 515 and 520 nm for nanoparticles of dimensions ranging from 15 to 20 nm and between 520 and 525 nm for nanoparticles of dimensions ranging from 20 to 30 nm, in perfect agreement with the experimental results obtained from AuNPs 1, AuNPs 2, and AuNPs 3. As far as AuNPs 4 is concerned, the calculated LSPR transversal component for nanoparticles of about 70 nm diameter is centered at around 600 nm, that is about 50 nm red-shifted with respect to the experimental value. This discrepancy can be explained by taking into account the increased size heterogeneity of the sample as well as the partial line shape overlapping of the two plasmonic band components.

### 3.2. Rheological Behavior of PVA-AuNP Suspensions

**Figure 3:**
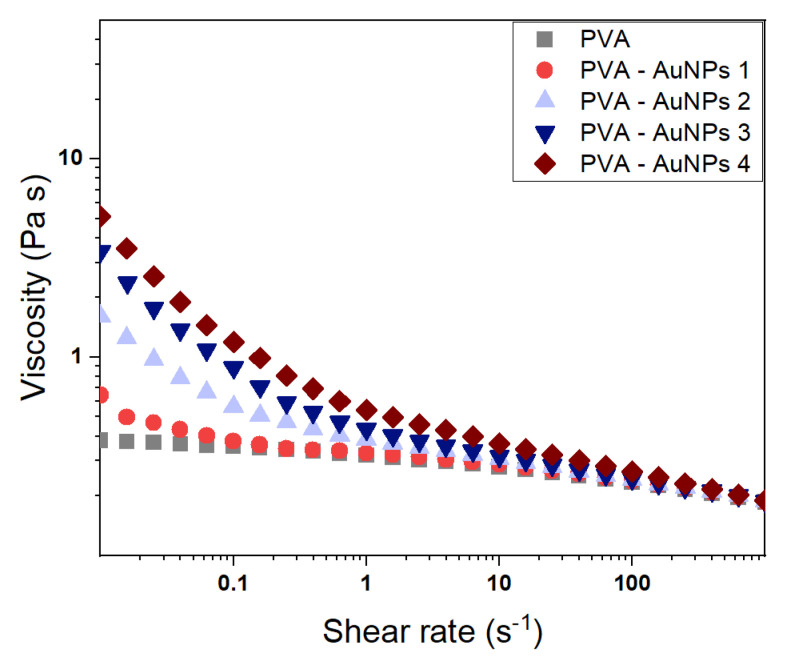
Steady-state viscosity curves of PVA-AuNPs suspensions measured at *T* = 25 °C.

The rheological properties of polymer-based suspensions containing nanoparticles and other nanostructures are of fundamental importance in determining both the system stability and processability. Generally speaking, nano-sized fillers dispersed in a liquid medium and surrounded by macromolecular chains can originate a transient secondary network via weak interactions thus modifying the overall rheological properties of the system. At the same time, the viscoelasticity provided by polymeric materials may stabilize the formulation hence preventing the coalescence and/or sedimentation of the nanofillers over time [37,38]. In particular, concerning the electrospinning technique, formulation viscosity and viscoelasticity play among others a topical role in affecting the nanofiber fabrication process. A low density of chain entanglements, which corresponds to a limited viscosity, usually leads to the breakage of the ejected polymer jet with the resultant formation of bead-like structures rather than homogeneous nanofibers. On the contrary, too high of a viscosity may prevent the maintenance of a constant infuse rate of the feeding mixture through the spinneret [39,40]. With these premises, the rheological properties of the prepared PVA-based mixtures were assessed to explore both the sample stability and suitability for the electrospinning technique. The resultant steady-state viscosity curves measured at *T* = 25 °C are shown in Figure 3.

The PVA solution (i.e., gray symbols) is characterized by the typical shear-thinning behavior of polymeric fluids with a constant viscosity at a low shear rate (i.e., Newtonian region) followed by a decrease of the viscosity as the shear rate is increased. Such a characteristic is commonly associated with the chain disentanglement induced by the applied shear and the related orientation of the macromolecules, which in turn reduces the polymer network density [41,42,43]. Generally speaking, beyond the viscosity value at rest (i.e., zero-shear viscosity η_0_), a marked shear-thinning is indicative of a high number of entanglements and may hence suggest the sample suitability for the electrospinning process. Concerning the PVA-AuNPs suspensions, as expected, they behave as Bingham fluids which are characterized by a high viscosity at rest but can easily flow once subjected to a certain stress (i.e., yield stress) that depends on the fluid composition [44]. Despite such behavior being observed independently of the AuNPs type, their size and anisotropy significantly affect the sample’s overall viscosity. In greater detail, the suspension viscosity at rest mainly grows as the nanoparticle’s dimensions increase (i.e., brown symbols vs. red symbols). At comparable nanoparticles size, the η_0_ value seems to also increase as the colloid anisotropy increases (i.e., violet symbols vs. blue symbols). However, it is noteworthy that the nanoparticle effect is progressively nullified by increasing the shear rate, with the formulations presenting more or less the same final viscosity. Hence, since the nanofiber formation during electrospinning occurs at very high shear rate values, the nanofiller presence is expected to have a negligible effect on the overall process viscosity dependence, as demonstrated by the successful fabrication of electrospun AuNPs-containing mats described in the following sections, but may influence the resultant mat morphology.

### 3.3. UV-vis-NIR Spectra of Electrospun Membranes

**Figure 4:**
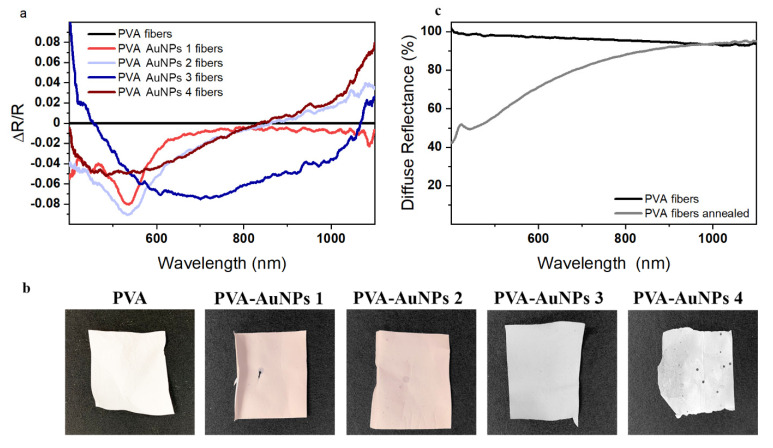
(**a**) Diffusive reflectance spectra of the as-prepared PVA-based membranes, (**b**) membrane digital photographs, and (**c**) comparison between the optical response before and after the thermal treatment for PVA fibers.

The presence of gold nanoparticles within the nanocomposite PVA-based fibers was firstly confirmed by means of diffuse reflectance spectroscopy measurements performed on the mats before the thermal annealing treatment. Figure 4a shows the different spectra measured for the four mats embedding the particles with respect to the pristine PVA fiber and calculated as (R-R_PVA_)/R_PVA_ in order to highlight the nanoparticle presence and absorbance. In this sense, all the fibers loaded with gold nanostructures show values of ΔR/R lower than zero in well-defined spectral intervals confirming the presence of absorbing species (see data in Figure 4a). In detail, PVA-AuNPs 1 and PVA-AuNPs 2 samples, which are loaded with the spherical particles, show a spectrally defined minimum peak at 535 nm, slightly red-shifted with respect to the absorbance peak observed for their analogous solution. Conversely, greater differences are detected for the other two samples with respect to their solution formulations (Figure 2). Specifically, when gold nanorods are loaded into the polymer matrix (i.e., PVA-AuNPs 3), the ΔR/R signal shows a broad negative signal in a wide spectral range from 450 to 1060 nm. Similarly, PVA-AuNPs 4 displays a broad negative and asymmetrical signal with a minimum positioned just below 500 nm. Such an enlarged signal may be attributed to the partial aggregation of particles during the electrospinning process and nanofiber fabrication. Digital photographs of the prepared mats are reported in Figure 4b to highlight the changes in their physical appearance depending on the embedded nanoparticles. A clear agreement between the naked eye observed color and the diffuse reflectance spectra reported in Figure 4a is observable. Specifically, PVA-AuNPs 1 and PVA-AuNPs 2 display a light-red color, whereas PVA-AuNPs 3 and PVA-AuNPs 4 show a grayish-blue and grayish-white appearance, respectively. Finally, as expected, PVA fibers are markedly white. Above all, it can be safely assumed that these results confirm the nanoparticle presence within the electrospun membrane.

For the sake of completeness, Figure 4c reports the reflectance spectra of the pristine PVA nanofibers before (black line) and after (gray line) annealing. Interestingly, the thermal treatment generates a decrease in the diffuse reflectance in the visible spectral range that induces a yellowish color to the fibers, most likely due to the occurrence of molecular changes induced by macromolecular thermolysis [45,46]. As such, the signals corresponding to the absorbance of the AuNPs cannot be clearly distinguished in the annealed fibers.

### 3.4. Stabilization and Thermal Properties of Electrospun Membranes

**Figure 5:**
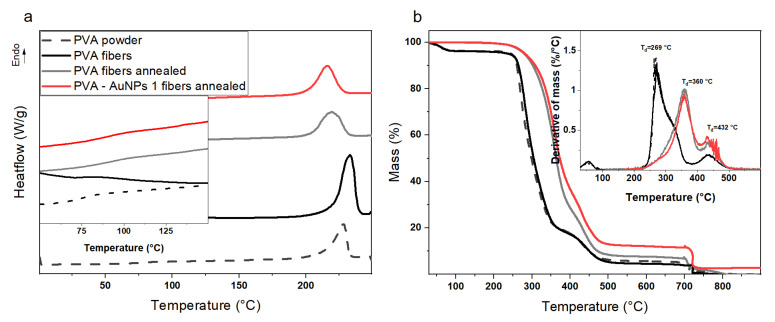
(**a**) DSC and (**b**) TGA thermograms of PVA powder, as-prepared PVA fibers, annealed PVA fibers, and annealed PVA-AuNPs 1 fibers. The inset in panel (**a**) reports a portion of the DSC curves to highlight the sample glass transition temperature, whereas the inset in panel (**b**) represents derivative thermogravimetric analysis (DTGA) curves.

Since PVA is a highly hydrophilic and water-soluble polymer, applying a proper stabilization treatment is of topical importance in order to avoid the material dissolution and/or structural modification (i.e., loss of the nanofibrous structure) in aqueous environments. In the present work, following a procedure reported in the literature [47,48,49], a simple, safe, and cheap thermal treatment carried out at *T* = 180 °C for 2 h was exploited to physically stabilized the nanofibers and avoid their rapid disintegration in contact with specific liquids. Differential scanning calorimetry was first used to explore the changes occurring in the sample upon the annealing treatment. Figure 5a and Table 2 report the DSC thermograms and thermal properties, respectively, of PVA powder, as-prepared PVA nanofibers, and annealed PVA nanofibers. The thermal profile and data obtained from annealed PVA-AuNPs 1 nanofibers, chosen as a reference sample to highlight the behavior of nanocomposite membranes, are also added.

PVA powder (dashed line) presents two thermal signals, one at T ~75 °C corresponding to the polymer glass transition temperature (*T*_g_) and one at T ~228 °C corresponding to the polymer melting temperature (*T*_m_). As-prepared PVA nanofibers (black line) are characterized by a very similar thermogram but melt at a slightly higher temperature with respect to the polymer powder. This result may be associated with the fact that during the electrospinning process the macromolecular chains are forced to assume a highly oriented conformation, which in turn increases the polymer melting resistance. However, as indicated by the normalized melt enthalpy values (Table 2), a reduction of PVA crystallinity can be noted after the electrospinning process and it is related to the extremely short time of solvent evaporation which hinders the crystallization process. Concerning annealed PVA nanofibers (gray line) and annealed PVA-AuNPs 1 nanofibers (red line), two distinct effects can be observed. Specifically, the glass transition occurs at *T*_g_ ~92 °C, which is well above that shown by the polymer powder and as-prepared nanofibers, and proves the stabilization effect of the annealing treatment. Furthermore, the polymer melting peak is displayed at a much lower temperature compared to the other two samples and it appears to be significantly wider. These differences may be attributed to the fact that during the thermal treatment at *T* = 180 °C changes in the arrangement of the macromolecules occur, during which some easily splitable polymer chains could even undergo chain scission [50,51]. Interestingly, it is noteworthy that the gold nanoparticle presence seems to further highlight such phenomenon since they can probably act as catalyzers. However, the annealing treatment seems in general to be able to increase the overall sample crystallinity that assumes values comparable to that of the polymer powder.

Along with DSC measurements, thermogravimetric analysis was carried out on the prepared PVA-based electrospun membranes to assess the sample thermal stability and effect of the annealing procedure. Figure 5b displays the TGA profiles of tested samples. PVA powder (dashed black line) and PVA nanofibers (black line) present the same thermal degradation kinetic. In particular, beyond an initial mass loss ascribable to the vaporization of residual humidity (i.e., T < 100 °C), a double step degradation can be observed with the related peaks centered at T_d,1_ ~269 °C and T_d,2_ ~432 °C followed by the complete disruption of the carbonaceous residue at T = 700 °C (i.e., switch of the fluxed gas from N_2_ to O_2_). Concerning the annealed PVA nanofibers (gray line), a considerable enhancement of the sample’s thermal stability can be depicted with respect to the as-prepared nanofibers. Specifically, along with the complete lack of residual humidity, the material appears to be stable up to T_1,d_ ~360 °C, whereas no noticeable differences are observed for the second degradation step (i.e., T_2,d_ ~432 °C). Hence, such a result confirms the efficiency of the annealing treatment in stabilizing PVA-based nanofibers as already shown by DSC measurements. Remarkably, annealed PVA-AuNPs 1 nanofibers are characterized by similar thermal behavior but present a final residual mass of around 3% corresponding to the percentage of gold nanoparticles effectively contained in the sample.

### 3.5. Morphological Properties of Electrospun Membranes

**Figure 6:**
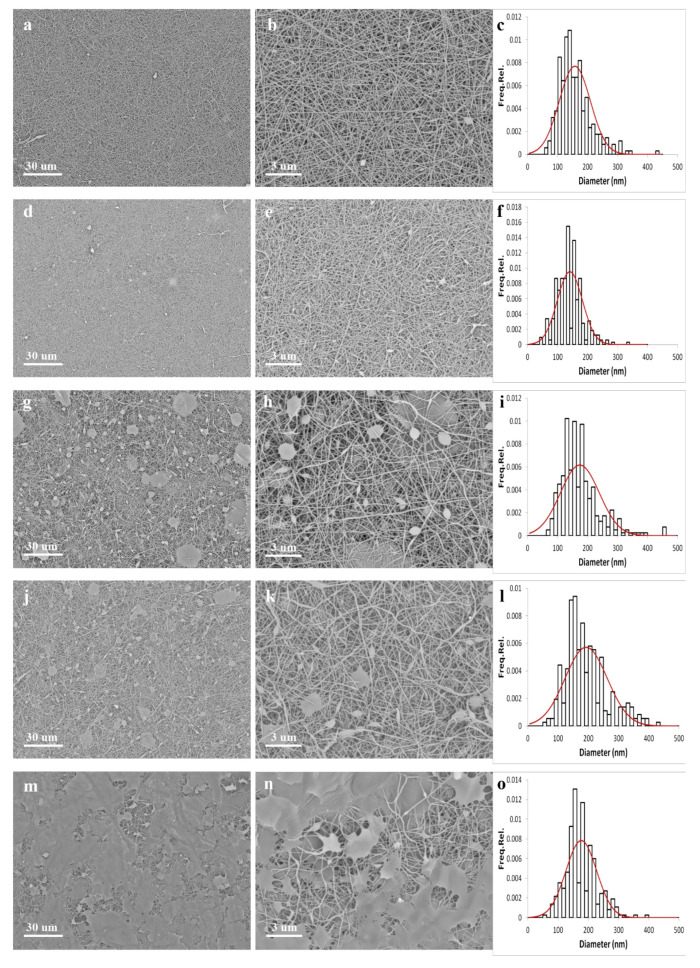
SEM micrographs and related nanofiber dimension distribution for (**a**–**c**) annealed PVA fibers, (**d**–**f**) annealed PVA-AuNPs 1 fibers, (**g**–**i**) annealed PVA-AuNPs 2 fibers, (**j**–**l**) annealed PVA-AuNPs 3 fibers, and (**m**–**o**) annealed PVA-AuNPs 4 fibers.

Electrospun membranes are usually composed of homogeneous sub-micron fibers organized in a nonwoven structure. This unique fibrous arrangement provides for a high area-to-volume ratio and a great porosity. These advantages, together with the fact that electrospun nanofibers can be used to embed nanoparticles and/or specific substances, make them extremely versatile materials for various applications [52,53]. However, it is noteworthy that the morphology of electrospun membranes, and the related physical-chemical properties, are controlled by the intercalation of several factors, namely the processing parameters (e.g., applied voltage, spinneret-collector distance, infuse rate), the environmental conditions (i.e., temperature and relative humidity), and the mixture properties (e.g., viscosity, conductivity, surface tension, polymer-solvent interactions, nanofiller content). Additionally, post-processing treatments (e.g., crosslinking, washing cycles) can also affect the nanofiber morphology and their impact should be taken into account. Figure 6 shows the SEM micrographs at two different magnifications, as well as the nanofiber size distributions, of the prepared PVA-based membranes after the thermal stabilization treatment.

The samples are characterized by a different nanofibrous architecture, but they do not show noticeable nanoparticle aggregates to indicate that the nanofillers are well dispersed within the polymeric fibers. Nonetheless, significant dissimilarities can be observed in terms of fiber dimensions and features of the porous structure. Table 3 summarizes the mat’s morphological results. In greater detail, PVA nanofibers (Figure 6a–c) and PVA-AuNPs 1 nanofibers (Figure 6d–f) display a striking well-defined morphology with homogeneous nanofibers showing diameters of 157 ± 52 nm and 141 ± 42 nm, respectively, and no clearly detectable defects. This slight decrease in the fiber dimension is most likely associated with the increased conductivity of PVA-AuNPs 1 formulation with respect to the PVA solution, which in turn favors the electrospinning process leading to thinner nanofibers. Concerning PVA-AuNPs 2 (Figure 6g–i) and PVA-AuNPs 3 (Figure 6j–l) samples, they are characterized by a still acceptable morphology consisting of quite homogeneous nanofibers with dimensions of 174 ± 65 nm and 193 ± 70 nm, respectively. Contrariwise to what was observed for PVA-AuNPs 1 nanofibers, these samples present a greater dimension with respect to the PVA reference. However, such a finding can be easily explained by considering the formulation of the rheological properties shown in Figure 3. Specifically, the significant increase of the viscosity shown by PVA-AuNPs 2 and PVA-AuNPs 3 mixtures likewise overcomes the conductivity effect, hence leading to greater nanofibers [54]. Furthermore, beyond the presence of bead-like defects, a few regions in which the nanofibrous structure is completely lost can be observed as well and are most likely due to the excess of CTAB used for nanoparticle synthesis. In this sense, the PVA-AuNPs 4 sample (Figure 6m–o) shows a strongly limited nanofibrous structure in favor of these “bulk” regions. However, the detectable nanofibers are quite homogeneous and present an average dimension of 177 ± 51 nm.

Generally speaking, it is noteworthy that only some of the prepared samples present a nanofibrous morphology and could be effectively used in practical applications. Specifically, PVA and PVA-AuNPs 1 fibers present a striking well-defined structure and they could be used in the present form without requiring the modification of the processing conditions. Yet, PVA-AuNPs 2 and AuNPs 3 are characterized by the presence of some defects, which, however, are most likely removable by optimizing the preparation procedure (i.e., nanoparticle concentration in the PVA-based formulations and electrospinning parameters). Conversely, PVA-AuNPs 4 shows a totally not satisfactory morphology with almost the complete loss of nanofibrous structure and it is hardly amendable, and it is consequently poorly suitable for any practical application.

As far as the porous structure of the mats is concerned, the nanocomposite samples show higher porosities with respect to their all-polymeric counterpart. Moreover, the porosity increases with increasing the nanoparticles size and anisotropy, thus resembling the trend already observed for the rheological properties of the corresponding suspensions. The average porosity values range from 12 to 16% and are very close to that (i.e., 14%) calculated for electrospun alginate membranes embedding zinc oxide nanoparticles reported in a previous work [22]. The analysis of the pore size shows that only macropores were formed in all the samples. Once again, the pore dimensions grow correspondingly with the steric hindrance of the embedded nanoparticles in the nanocomposite mats. The trend is particularly highlighted for the PVA-AuNPs 4 membrane, characterized by the presence of the largest macropores as well as the lowest percentage of macropores below 2 µm.

### 3.6. Mechanical Behavior of Electrospun Membranes

**Figure 7:**
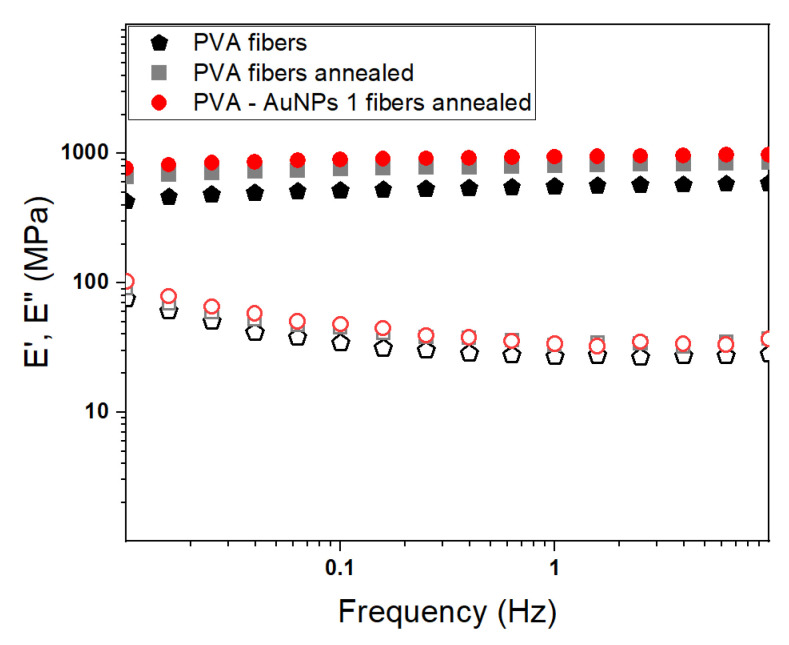
DMA spectrum of as-prepared PVA fibers, annealed PVA fibers, and annealed PVA-AuNPs 1 fibers. Filled and empty symbols represent the extensional storage and extensional loss moduli, respectively.

The mechanical properties of polymeric and composite materials are fundamental to establish their applicability for a specific purpose. Polymer-based electrospun membranes are usually characterized by a self-standing nature, a good mechanical resistance, and a marked foldability, thereby being appropriate in several application fields. In this regard, it is noteworthy that the mechanical response of electrospun nanofibers can be easily tuned by controlling their size and structural organization, by performing post-processing treatments, or by introducing in the structure reinforcement nanofillers as vastly reported in the literature [55,56]. Hence, in the present work dynamic-mechanical analysis was exploited to assess the sample’s overall mechanical response and evaluate the effect of both the annealing process and AuNPs’ presence. Once again, the mat embedding AuNPs 1 was chosen as a reference sample of annealed nanocomposite membranes. Figure 7 shows the DMA curves at T = 25 °C of PVA nanofibers, annealed PVA nanofibers, and annealed PVA-AuNPs 1 nanofibers. A summary of the mat’s mechanical properties (i.e., E′ and E″) is reported in Table 4.

Independently of the sample, the characteristic behavior of solid polymeric materials is depicted in the investigated frequency range and it corresponds to the rubbery plateau region. Specifically, the extensional storage modulus (E′) slightly increases as the frequency is raised, while the extensional loss modulus (E″) shows an opposite trend. Despite this, no significant differences can be observed among the samples in terms of their overall mechanical behavior, it is noteworthy that the annealing treatment seems to be able to increase the sample’s stiffness (i.e., Δ = 45%). Similarly, as already reported [57], the nanofillers can act as mechanical reinforcement leading to an enhanced mechanical response. In general, it can be safely stated that the prepared PVA-based electrospun membranes are characterized by good mechanical response and could be hence employed for several purposes.

## 4. Conclusions

In the present research work, composite PVA nanofibers embedding gold nanostructures prepared via a single-step electrospinning process were reported. To this purpose, both spherical nanoparticles (i.e., AuNPs 1 and AuNPs 2) of average dimensions of about 13 and 15 nm and nanorods (i.e., AuNPs 3 and AuNPs 4) with a different diameter (about 15 and 70 nm, respectively) and aspect ratio (about 2.4 and 1.7, respectively) were obtained by properly adjusting wet synthetic protocols. Specifically, the effect exerted by the nanofiller size and anisotropy on the fiber properties was investigated. These nanostructures were hence dispersed in PVA aqueous solutions, which turned out to exhibit a progressively more marked Bingham fluid behavior with the increasing of the colloid steric hindrance, and were successfully electrospun to prepare nanofibrous mats. The fabricated meshes were then stabilized via a simple thermal treatment (i.e., T = 180 °C for 2 h) to induce PVA physical crosslinking, as demonstrated by means of DSC and TGA characterization. Specifically, such a treatment considerably improved the physical-chemical properties of the samples, such as crystallinity, thermal stability up to 350 °C, and water resistance, particularly profitable for their practical use. Additionally, the composite mats displayed a self-standing nature and good mechanical resistance (i.e., E′ = 556–945), with the latter being further enhanced by both the nanoparticle’s presence and thermal annealing. As far as the mat morphology is concerned, changes in the overall sample morphology (i.e., homogeneity and porosity) were observed as a function of the nanofiller type. Specifically, the smaller and more isotropic the embedded nanostructures, the thinner and more defined the resultant PVA-based nanofibers.

Generally speaking, the proposed fabrication approach was demonstrated as a straightforward single-step methodology to develop composite PVA-based nanofibrous meshes with relatively high porosity, water insolubility, mechanical robustness, and thermal resistance. In this sense, the presence of gold nanostructures with tailor-made morphology and plasmonic features may allow using the proposed composite materials for a broad range of applications, including the development of sensors for wastewater, air pollution, and even for specific biomolecules.

## Figures and Tables

**Table 1:** Summary of the nanostructure morphological and optical properties.

Sample	Diameter (nm)	Length (nm)	Aspect Ratio	Absorbance Peak (nm)
AuNPs 1	13.4 ± 2.9	13.4 ± 2.9	1	521
AuNPs 2	15.5 ± 4.9	15.5 ± 4.9	1	524
AuNPs 3	15.9 ± 3.5	36.5 ± 6.3	2.4 ± 0.6	524–686
AuNPs 4	71.4 ± 18.8	105.6 ± 25.5	1.7 ± 0.7	552–669

**Table 2:** Thermal properties and crystallinity degree of PVA powder, as-prepared PVA fibers, annealed PVA fibers, and annealed PVA-AuNPs 1 fibers.

Sample	T_g_ (°C)	T_m_ (°C)	ΔH_m_ (J/g)	Δ_c_ (%)	T_d,1_ (°C)	T_d,2_ (°C)
PVA powder	75	228	92.3	67	268.6	431.9
PVA fibers	74	233	87.1	63	269.1	432
Annealed PVA fibers	92	218	93.5	67	360	431.8
Annealed PVA-AuNPs 1 fibers	92	215	93.4	67	359.9	432.1

**Table 3:** Summary of the electrospun membrane’s morphological properties.

Sample	Fiber Diameter (nm)	Porosity (%)	Pore Size (µm)	Pore Size < 0.2 µm (%)
PVA	157 ± 52	11.9	0.1–1.1	65
PVA-AuNPs 1	141 ± 42	12.2	0.1–1.1	70
PVA-AuNPs 2	174 ± 65	13	0.1–2.1	60
PVA-AuNPs 3	193 ± 70	14.1	0.1–1.8	59
PVA-AuNPs 4	177 ± 51	15.8	0.1–2.9	31

**Table 4:** Dynamic-mechanical properties of as-prepared PVA fibers, annealed PVA fibers, and annealed PVA-AuNPs 1 fibers. Moduli values have been taken at a frequency of 1 Hz.

Sample	E′ (MPa)	E″ (MPa)
PVA fibers	556 ± 13	27 ± 2
Annealed PVA fibers	808 ± 21	33 ± 2
Annealed PVA-AuNPs 1 fibers	945 ± 9	34 ± 1

## Data Availability

Data will be made available on request.
